# Occlusal Force Changes in Growing Patients Treated with the Three Bite Plane Appliance: A Prospective Study

**DOI:** 10.3390/dj14040198

**Published:** 2026-04-01

**Authors:** Mauro Lorusso, Angela Pia Cazzolla, Michele Tepedino, Elena D’Angelo, Fariba Esperouz, Lucio Lo Russo, Domenico Ciavarella

**Affiliations:** 1 Department of Clinical and Experimental Medicine, Dental School of Foggia, University of Foggia, 71122 Foggia, Italy; elena.dangelo@unifg.it (E.D.); fariba.esperouz@unifg.it (F.E.); lucio.lorusso@unifg.it (L.L.R.); domenico.ciavarella@unifg.it (D.C.); 2 Department of Medicine and Surgery, Lum University, 70010 Casamassima, Italy; cazzolla@lum.it; 3 Department of Biotechnological and Applied Clinical Sciences, Dental School of L’Aquila, University of L’Aquila, 67100 L’Aquila, Italy; michele.tepedino@univaq.it

**Keywords:** occlusal force, deep-bite, three-bite plane appliance, growing patients

## Abstract

**Objective:** To investigate the effects of three-bite plane appliance (TBP) therapy on maximal occlusal force (MOF) in growing patients. **Methods:** This study included 120 children (aged 9–10 years) diagnosed with Class I, Class II, or Class III malocclusion. All subjects presented with a deep bite and a normodivergent growth pattern. MOF was recorded at baseline (T0) and after 12 months of treatment (T1). A standardized multi-bite protocol was used to improve reproducibility. Paired t-tests or Wilcoxon tests assessed intragroup differences, while Welch’s ANOVA with Games–Howell post hoc testing evaluated intergroup variation. **Results:** Significant intragroup differences were found for all groups (*p* < 0.001). Class I subjects demonstrated a reduction in MOF (Δ = −138.3 N; *p* < 0.001), whereas Class II (Δ = 113.35 N; *p* < 0.001) and Class III (Δ = 145.6 N; *p* < 0.001) subjects showed significant increases. Intergroup comparison revealed a significant overall difference in MOF change (F = 41.35; *p* < 0.001). Post hoc analysis confirmed significant differences between Class I and both Class II and III, while no significant difference was detected between Class II and III. **Conclusions:** Treatment with a three-bite plane appliance modifies MOF in growing patients, showing malocclusion-specific adaptation patterns. The reduction observed in Class I contrasts with the functional enhancement detected in Class II and Class III subjects.

## 1. Introduction

Dental occlusion refers to the spatial and functional relationship between the maxillary and mandibular dentition, integrating the coordinated activity of the masticatory muscles and the temporomandibular joint (TMJ). It is traditionally divided into static and dynamic components, both of which contribute to maintaining orofacial equilibrium and ensuring efficient load transmission across the craniofacial complex [1]. Static occlusion describes the tooth contacts that occur when the mandible is fully closed and stationary, typically in the maximum intercuspal position (MIP). In this highly reproducible position, the dental arches establish a stable occlusal relationship characterized by multiple, firm contact points that facilitate a balanced distribution of occlusal forces. Any deviation from the ideal static occlusal scheme, such as premature contacts, interferences, or an unstable intercuspal position, may predispose individuals to abnormal loading patterns, enamel wear, periodontal strain, and occlusal trauma. Dynamic occlusion, conversely, concerns the tooth contacts generated during functional mandibular movements including protrusion, retrusion, and lateral excursions [2]. These movements require precise coordination between the dental guidance systems, neuromuscular activity, and the biomechanics of the TMJ [3]. During protrusive movements, the anterior teeth, primarily the incisors, guide the mandible forward while disengaging the posterior dentition, thereby minimizing horizontal loads on molars and premolars. In lateral excursions, guidance may be provided by the canines (canine guidance) or by multiple posterior teeth on the working side (group function). Although both patterns can be physiologic, their efficiency depends on the absence of harmful interferences that might alter mandibular kinematics. Non-working-side interferences, for example, are recognized as detrimental because they may increase elevator muscle activity, modify chewing patterns, and impose excessive stresses on the periodontal ligament and temporo-mandibular joint disorders (TMJDs) [4]. Such dysfunctions may contribute to parafunctional habits, altered muscle recruitment strategies, occlusal instability, and the development or perpetuation of TMJDs.

Occlusal force represents the resultant load exerted on the dentition through the activity of the masticatory muscles during both functional tasks such as mastication, swallowing, and speech and parafunctional activities, including bruxism and clenching [5]. As an integrated indicator of masticatory muscle performance, occlusal force reflects the combined influence of neuromuscular coordination, TMJ function, dentoalveolar morphology, and skeletal architecture [6,7]. Numerous biological and environmental factors modulate its magnitude and direction, including craniofacial growth patterns, sexual dimorphism, age-related muscular development, airway patency, dental arch form, and occlusal morphology [8,9]. These variables are particularly dynamic during growth, where physiological adaptations and environmental influences interact to shape the developing occlusal system.

In this context, respiratory mode has been shown to exert a significant effect on orofacial growth and function. Masutomi et al. [10] demonstrated that adolescents with chronic mouth breathing exhibit distinctive morphological characteristics such as posterior positioning of the hyoid bone and clockwise mandibular rotation along with reduced functional capabilities including lip-closing force, tongue pressure, and masticatory efficiency. These findings underline the importance of orofacial muscular forces in guiding skeletal and dental development. Similarly, vertical and sagittal craniofacial growth patterns influence dental arch form, occlusal contacts, and the maxillomandibular spatial relationship, thereby affecting occlusal force distribution during function [11,12]. Genetic or congenital conditions such as cleft lip and palate may further modify occlusal relationships by altering arch morphology, muscular function, and airway dynamics, with potential impacts on swallowing and breathing mechanisms [13,14,15].

In growing subjects, maximum occlusal force is a particularly relevant parameter because it reflects the combined effects of craniofacial growth, occlusal development, and neuromuscular maturation. During this phase, changes in skeletal and dentoalveolar relationships may influence the functional expression of bite force. Therefore, its evaluation may offer useful background information for interpreting the functional adaptation of the stomatognathic system during orthodontic and orthopedic treatment.

Orthodontic and orthopaedic interventions during growth can play a crucial role in modulating occlusal forces and guiding the development of a stable occlusion. Functional appliances can modify mandibular posture, neuromuscular patterns, and vertical skeletal relationships, thereby influencing the distribution of occlusal forces and improving the efficiency of the masticatory system. Clear aligners have emerged as an alternative option capable of addressing vertical discrepancies and exerting controlled effects on the occlusal plane [16,17]. More recently, the advent of direct-printed aligners has introduced novel biomechanical considerations, as their material properties and force delivery characteristics differ from those of conventional thermoformed appliances, necessitating further investigation into their effects on occlusion and force transmission [18].

Maximum occlusal force is a relevant functional parameter for evaluating the performance of the stomatognathic system and requires measurement tools with adequate reliability and clinical applicability. Among the available digital devices, the Innobyte system has recently been investigated in different populations and clinical settings. In particular, Ustrell-Barral et al. [19] specifically assessed the device and reported excellent reliability, providing reference values for maximum bite force measured with Innobyte in healthy adults. In addition, the same system has been used in growing patients with posterior crossbite [9], in healthy adults in relation to sleep bruxism and masticatory performance [20], and in older adults in studies on oral motor function [21]. Taken together, these findings support the reproducibility of the Innobyte system and its applicability for clinical occlusal force assessment across different age groups and clinical conditions.

Beyond its biomechanical significance, maximum occlusal force may also represent a clinically useful functional marker of adaptation during growth and orthodontic treatment. In growing subjects, the evaluation of bite force may provide indirect information on the interaction between occlusal relationships, neuromuscular coordination, and the functional response of the stomatognathic system to treatment. From a clinical perspective, monitoring occlusal force may help to better interpret treatment-related changes in masticatory performance and functional reorganization, particularly in patients undergoing orthopedic correction of sagittal discrepancies. For this reason, the assessment of maximum occlusal force may have potential applications not only for research purposes, but also for the functional monitoring of treatment progression in daily clinical practice.

Several devices have been developed to assess occlusal force, either across the entire dental arch or within specific unilateral regions. Among the systems most commonly used in clinical and research settings are Dental Prescale II (GC Corp., Tokyo, Japan), which provides quantitative force values, and T-Scan (Tekscan Inc., Norwood, MA, USA), which mainly records relative force distribution [22,23]. More recently, Innobyte has been introduced as a portable device for bite-force assessment. Equipped with a pressure sensor, it enables the evaluation of both whole-arch and unilateral occlusal force and provides a direct quantitative readout in newtons. This feature may be particularly useful when assessing patients expected to generate high occlusal loads and when planning complex implant–prosthetic rehabilitations.

Given the complex interplay between occlusal forces, craniofacial growth, and neuromuscular regulation, even minor deviations from physiological loading patterns may influence occlusal stability, periodontal health, TMJ function, and long-term adaptability [24,25]. Understanding these relationships is essential, especially in growing patients undergoing orthodontic or functional orthopaedic treatment, where therapeutic modulation of occlusal forces may enhance treatment outcomes and promote the development of a stable, harmonious, and functionally efficient occlusion. The aim of the present study was to assess changes in occlusal force after functional therapy using the three-bite appliance (TBP) in growing patients.

## 2. Material and Methods

The study followed the recommendations of the Strengthening the Reporting of Observational Studies in Epidemiology (STROBE) statement [26]. All procedures outlined in the study protocol adhered to the principles of the Declaration of Helsinki and were approved by the Ethics Committee of the University.

The sample consisted of three groups of patients: 40 with Class I malocclusion (mean age 9.4 ± 0.4), 40 with Class II malocclusion (mean age 9.5 ± 0.1), and 40 with Class III malocclusion (mean age 9.7 ± 0.2). The inclusion criteria were as follows: patients presenting with Class I, II, or III malocclusion; an age range of 9–10 years; a normodivergent facial pattern (30.5° ≤ SN-GoMe ≤ 35.5°); the presence of a deep bite with increased overbite; and patients treated with TBP. Patients were excluded if they exhibited tooth agenesis, periodontal disease, a history of orthodontic treatment, early loss of one or more teeth, open bite, congenital craniofacial anomalies, or a fully permanent dentition. Cephalometric analysis was performed by an experienced orthodontist and the variables analyzed are shown in Table 1.

A sample size calculation was carried out using G*Power (version 3.1.9.2; Franz Faul, University of Kiel, Kiel, Germany). Assuming an effect size of 0.4 [27], a one-way ANOVA, an α level of 0.05, and a statistical power of 0.95, the analysis indicated that at least 34 participants per group were required.

### 2.1. Measuring Device

**Figure 1:**
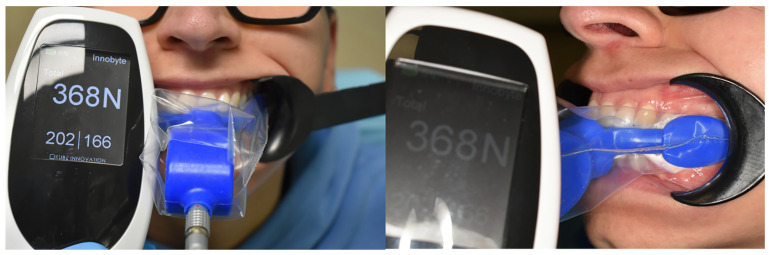
Innobyte measurements.

The Innobyte system is a medical device designed to assess occlusal force and to record maximum bite force either for the entire dental arch or separately for each side. It is composed of two parts: a main unit and an intraoral mouthpiece. The system measures occlusal force within a range of 0 to 2000 N, with a reported accuracy of 5%. The mouthpiece is filled with fluid, allowing the applied biting pressure to be distributed through the medium and detected by the internal sensors. The recorded values are displayed on the main unit through an LED screen, with force expressed in Newtons. For each measurement, the device provides the overall occlusal force as well as the corresponding left and right-side values. Figure 1 shows MOF measurements obtained with the Innobyte.

### 2.2. Measurement Protocol

The study procedures were explained to all participants, and informed consent was obtained from their parents. Occlusal force was evaluated at baseline (T0) and after 12 months of treatment with the TBP (T1) using the Innobyte device (kube innovation, Montreal, QC, Canada).

Each patient was seated in the dental chair with the head in a natural position and the mandible parallel to the floor. The silicon pad was placed intraorally between the dental arches, ensuring that the central notch aligned with the patient’s maxillary and mandibular central incisors. Data acquisition was initiated by pressing the activation button, and the patient was instructed to perform a maximal voluntary clench. After each force measurement, the mouthpiece was removed and the patient was asked to swallow. Following swallowing, the patient maintained a rest position for 10 s before the next recording. To reduce measurement variability, a multibite protocol was used, consisting of three consecutive recordings for each subject [28].

#### Three-Bite Plane Appliance

The TBP is a removable functional orthodontic device comprising an anterior bite plane and two posterior bite planes connected by a central expansion screw. The appliance is fitted with an external labial bow incorporating two adjustment loops at the canine level and retention hooks on the maxillary first molars. Patients were instructed to wear the appliance during night-time and afternoon hours, for an average duration of approximately 14 h per day. Patient compliance was monitored through scheduled monthly follow-up visits. In particular, the presence of the characteristic gothic arch wear pattern on the metallic bite surfaces of the appliance produced by repeated mandibular functional movements was used as an indicator of regular appliance use. Based on these clinical observations, patients were considered generally compliant with the prescribed use of the devices.

**Figure 2:**
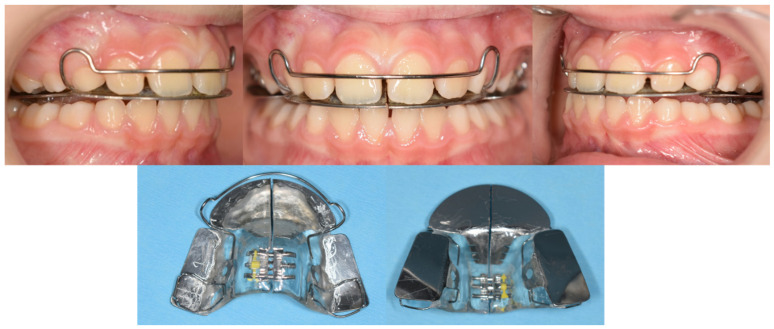
Intraoral photographs and frontal views of the three-bite plane (TBP) appliance.

Figure 2 illustrates the frontal view of the TBP and its intraoral positioning.

### 2.3. Statistical Analysis

Data were analysed using GraphPad Prism software 6.0 (GraphPad Prism Software, San Diego, CA, USA). Table 2 presents the occlusal force values for the analyzed groups before and after treatment and the Shapiro–Wilk test was performed to assess data distribution. To evaluate the treatment effect and compare pre- and post-intervention values within each sample group, a paired-sample t-test was used (Table 3). In cases of non-normal distribution, the Wilcoxon test was applied. Since the assumption of equal variances was violated, Welch’s ANOVA test (Table 4) followed by Games–Howell post hoc test (Table 5) was employed to compare T1–T0 changes in total force among the three groups.

## 3. Results

All three malocclusion groups exhibited significant intragroup modifications in maximal occlusal force: Class I demonstrated a marked post-treatment reduction (Δ = −138.3 N; *p* < 0.001), whereas Class II and Class III exhibited significant increases (Δ = 113.35 N and 145.6 N, respectively; *p* < 0.001). Due to violation of the homoscedasticity assumption, intergroup comparisons of treatment-induced changes (T1–T0) were performed using Welch’s ANOVA, which revealed a robust group effect (*F* = 41.35; *p* < 0.001). Post hoc analysis using the Games–Howell procedure showed that Class I differed significantly from both Class II and Class III, confirming heterogeneity in force modulation patterns among malocclusion types. Conversely, the pairwise comparison between Class II and Class III did not reach statistical significance (mean difference = −32.25 N; *p* = 0.07).

## 4. Discussion

The present study evaluated changes in maximal occlusal force (MOF) in growing patients treated with the TBP, stratified by sagittal malocclusion. A clear malocclusion-dependent trajectory emerged: Class I patients exhibited a significant reduction in MOF, whereas Class II and Class III patients showed substantial increases over the 12-month treatment period. Welch’s ANOVA confirmed robust inter-group differences, with post hoc testing indicating significant contrasts between Class I and both Class II and III, while Class II and III did not differ significantly. These findings reinforce the concept that functional orthopedic therapy modulates neuromuscular performance through mechanisms that are closely aligned with the underlying malocclusion. The decline in MOF documented in Class I subjects is consistent with recent evidence demonstrating that orthodontic treatment frequently induces transient reductions in masticatory function. Systematic review have confirmed that bite force decreases during the early phases of orthodontic therapy, largely due to pain, occlusal instability, and periodontal ligament sensitization that compromise neuromuscular output [29,30]. Mixed-dentition studies further show that occlusal alterations disrupt sensorimotor integration and reduce masticatory effectiveness during active treatment [31]. Deep-bite correction typically entails temporary reductions in anterior occlusal support, altering the vertical dimension and reducing the mechanical advantage of the elevator muscles. These changes may attenuate periodontal mechanoreceptor feedback, a key regulator of bite force, until a new neuromuscular equilibrium is achieved. Recent studies have demonstrated that bite force is highly sensitive to occlusal contact area, intercuspation quality, and dental-stage related proprioceptive changes [28,32]. Thus, the reduction in MOF observed in Class I subjects likely reflects a transient adaptive response rather than structural or functional impairment.

In contrast, Class II and Class III subjects exhibited significant increases in MOF showing that functional appliances can enhance masticatory performance when sagittal discrepancies impair baseline neuromuscular biomechanics. Mandibular advancement in Class II patients has been shown to improve length–tension relationships of the elevator muscles and optimize condylar position, resulting in increased bite-force capacity [33]. Several recent clinical trials have confirmed that fixed functional appliances, including Forsus and other sagittal correctors, significantly increase bite force, masticatory efficiency, and occlusal load distribution during growth [34,35]. Recent evidence has further highlighted the importance of dento-skeletal adaptations and vertical dimension control during orthodontic treatment in growing patients. Paoloni et al. [36] demonstrated that different expansion protocols in prepubertal subjects can produce distinct dento-skeletal responses, emphasizing the role of appliance design in modulating craniofacial adaptation during growth. Similarly, Lione et al. [37] reported that orthodontic appliances used for sagittal correction may influence vertical dimension and occlusal relationships in growing individuals. These findings support the concept that orthodontic and functional appliances can induce complex biomechanical and neuromuscular adaptations of the stomatognathic system during growth, which may also contribute to the changes in occlusal force observed in the present study.

Functional appliances are known to influence not only skeletal relationships but also the neuromuscular balance of the stomatognathic system [38]. Mandibular repositioning during growth can modify muscle recruitment patterns and improve functional coordination between the masticatory muscles and occlusal contacts [33]. These adaptive responses may contribute to the improvement in bite-force generation observed after sagittal correction.

In Class III malocclusion, pre-treatment occlusal instability and anterior dental interferences often lead to inefficient neuromuscular compensation. Correction of sagittal relationships improves intercuspation and reduces functional displacement, allowing more physiological activation of the masticatory musculature. Recent 3D occlusal analyses demonstrate that sagittal and vertical occlusal features are strongly associated with masticatory performance and bite force in young children [39], while functional rehabilitation in skeletal discrepancies has been shown to enhance neuromuscular efficiency [19]. Bite force naturally increases throughout childhood and adolescence. Recent studies show progressive enhancement in maximum bite force, with distinctive sex-related patterns and physiologic plateaus emerging during late adolescence [40]. Additional work has demonstrated that maturation of tongue pressure, lip strength, occlusal contact area, and masticatory efficiency follows coordinated developmental trajectories that significantly influence MOF [41]. Thus, the increases in MOF observed in Class II and III subjects likely reflect a synergy between treatment-induced occlusal improvement and inherent developmental maturation of the craniofacial and neuromuscular system.

Another factor that may contribute to the observed changes in MOF is the improvement in occlusal contact distribution achieved during treatment. Previous studies have demonstrated that bite force is strongly associated with the number and stability of occlusal contacts, as well as with the quality of intercuspation [42]. Functional treatment that improves sagittal relationships may therefore indirectly enhance bite-force generation by increasing occlusal support and mechanical efficiency during clenching [43].

The lack of a statistically significant difference between the MOF increments recorded in Class II and Class III patients is consistent with evidence showing that once sagittal discrepancies are corrected and intercuspation improves, children approach a functional ceiling for bite-force generation [40]. This plateau likely reflects the interaction of muscular architecture, craniofacial geometry, and available occlusal support. Individual variability arising from growth velocity, dental-stage progression, and neuromuscular maturation may further contribute to the overlapping post-treatment trajectories.

The divergent MOF trajectories observed across malocclusions carry significant clinical implications. In Class I deep-bite patients, reduced MOF may act as a protective adaptation by lowering functional loading during phases of occlusal reorganization, thereby mitigating strain on anterior teeth, periodontal tissues, and the temporomandibular joint. In Class II and III subjects, increases in MOF most likely reflect enhanced masticatory effectiveness and improved neuromuscular integration following sagittal correction. Recent evidence shows that increased masticatory efficiency and occlusal load distribution after functional therapy correlate with improved long-term stability of orthopedic and orthodontic outcomes [34,44]. Given the strong association between MOF and neuromuscular function, incorporating bite-force assessment into functional treatment protocols may provide valuable insight into treatment progression and functional adaptation. MOF may serve as a sensitive adjunctive biomarker to evaluate occlusal stability and neuromuscular integration during active growth and in post-treatment phases.

Baseline differences in maximum occlusal force were observed among the study groups and should be considered when interpreting the results. Such initial variability may have affected the magnitude of the changes observed during treatment. Consequently, part of the differences detected over time may reflect the heterogeneity of baseline values rather than treatment effects alone, and the findings should therefore be interpreted with appropriate caution.

Future research should further clarify the role of maximal occlusal force as a clinically relevant functional parameter for the assessment of occlusal neuromuscular function during growth and orthodontic treatment. Because bite force reflects the interaction between muscle recruitment, occlusal contacts, intercuspation, and craniofacial morphology, its measurement may provide useful indirect information on the functional adaptation of the stomatognathic system beyond morphologic correction alone. In this perspective, the routine assessment of maximal occlusal force could help identify changes in neuromuscular efficiency during treatment, monitor the establishment of a more stable occlusal balance, and improve the functional interpretation of treatment outcomes. Future studies should therefore investigate the relationship between bite-force values and other indicators of neuromuscular function, such as electromyographic activity, masticatory efficiency, and dynamic occlusal parameters, in order to validate occlusal force as a reliable biomarker of functional adaptation in growing orthodontic patients.

### Limitations of the Study

Occlusal force was evaluated under static clenching conditions, without incorporating dynamic functional parameters such as chewing efficiency, contact timing, or force distribution. Neuromuscular adaptation was not directly examined, as no electromyographic assessment was included. The sample was limited to normodivergent deep-bite children in mixed dentition, which restricts the generalizability of these findings to other craniofacial phenotypes or vertical skeletal patterns. Another limitation of the present study is the absence of a growth-matched untreated control group. Because maximum occlusal force is known to increase during normal growth and maturation of the masticatory system, the observed changes cannot be entirely attributed to the treatment and may partially reflect physiological developmental processes.

Future studies should incorporate electromyographic assessments to characterize changes in muscle recruitment following sagittal correction, as well as 3D occlusal contact analyses to elucidate how alterations in intercuspation affect force dynamics.

## 5. Conclusions

This study reports that functional bite-appliance therapy during growth can substantially modulate occlusal force, with distinct responses across malocclusion types. The reduction in MOF observed in Class I patients, contrasted with the increases recorded in Class II and Class III patients, likely reflects differential neuromuscular adaptation and occlusal reorganization. However, occlusal force was assessed under static clenching conditions; therefore, the findings should be interpreted within the limits of this specific measurement.

These findings highlight the need for individualized treatment planning that accounts for skeletal and dentoalveolar morphology, growth pattern, and functional potential to optimize therapeutic outcomes. A more comprehensive understanding of occlusal force adaptation may ultimately enhance long-term stability and functional performance in orthodontic patients.

## Figures and Tables

**Table 1:** Cephalometric characteristics of the sample.

SAMPLE	ANB	SN-GoMe	OVERBITE
CLASS I MALOCCLUSION (*n* = 40)	2.2 ± 0.4	31.1 ± 0.5	4.3 ± 0.4
CLASS II MALOCCLUSION (*n* = 40)	4.5 ± 0.5	31.3 ± 0.4	4.6 ± 0.3
CLASS III MALOCCLUSION (*n* = 40)	0.5 ± 0.4	31.4 ± 0.7	4.2 ± 0.3

**Table 2:** Descriptive statistics.

SAMPLE	OCCLUSAL FORCE (MEAN ± SD)	MEDIAN	MINIMUM	MAXIMUM	PASSED NORMALITY TEST?
CLASS I (T0)	690.4 ± 141.6 N	697 N	456 N	901 N	YES
CLASS I (T1)	552.1 ± 111.5 N	562.5 N	390 N	760 N	YES
CLASS II (T0)	266.25 ± 76.9 N	292.5 N	137 N	371 N	NO
CLASS II (T1)	379.6 ± 8.9 N	389.5 N	243 N	509 N	YES
CLASS III (T0)	189.3 ± 77.9 N	167 N	110 N	353 N	NO
CLASS III (T1)	334.9 ± 70.4 N	299.5 N	279 N	501 N	NO

**Table 3:** Pre- and post-treatment comparison: paired t-test or Wilcoxon signed-rank test.

SAMPLE	T1–T0 DIFFERENCE	IC95%	t	GL	*p*
CLASS I	−138.3	76.54, 200.05	4.68	39	<0.001
CLASS II	+113.35	−135.67, −91.02	−10.62	39	<0.001
CLASS III	+145.6	−165.21, −126.98	−15.53	39	<0.001

**Table 4:** Welch’s ANOVA used to assess T1–T0 differences in occlusal force among the groups.

TEST	gl1	gl2	F	*p*
Welch-Anova	2	34.75	41.35	<0.001

**Table 5:** Games–Howell post hoc test.

Dependent Variable	(I) Group	(J) Group	Mean Difference (I − J)	Std Error	Sig.	95% Confidence Interval	
						Lower Bound	Upper Bound
TOTAL FORCE	I	II	−251.65	31.37	0.0001	−330.02	−173.28
	I	III	−283.9	30.58	0.0001	−361.48	−206.32
	II	III	−32.25	14.2	0.07	−66.9	−2.4

## Data Availability

The data presented in this study are available on request from the corresponding author due to privacy reasons.
